# A data science approach for the classification of low-grade and high-grade ovarian serous carcinomas

**DOI:** 10.1186/s12864-018-5177-9

**Published:** 2018-11-27

**Authors:** Sangdi Lin, Chen Wang, Shabnam Zarei, Debra A. Bell, Sarah E. Kerr, George C. Runger, Jean-Pierre A. Kocher

**Affiliations:** 1 Department of Health Sciences Research, Mayo Clinic, Rochester, 55905 MN USA; 2 School of Computing, Informatics, and Decision Systems Engineering, Arizona State University, Tempe, 85281 AZ USA; 3 Department of Biomedical Informatics, Arizona State University, Scottsdale, 85259 AZ USA; 4 Department of Laboratory Medicine and Pathology, Mayo Clinic, Rochester, 55905 MN USA

**Keywords:** Copy number alternations, Low-coverage whole genome sequencing, Ovarian serious carcinoma, Machine learning, Data science, Tumor grade, Classification

## Abstract

**Background:**

Copy Number Alternations (CNAs) is defined as somatic gain or loss of DNA regions. The profiles of CNAs may provide a fingerprint specific to a tumor type or tumor grade. Low-coverage sequencing for reporting CNAs has recently gained interest since successfully translated into clinical applications. Ovarian serous carcinomas can be classified into two largely mutually exclusive grades, low grade and high grade, based on their histologic features. The grade classification based on the genomics may provide valuable clue on how to best manage these patients in clinic. Based on the study of ovarian serous carcinomas, we explore the methodology of combining CNAs reporting from low-coverage sequencing with machine learning techniques to stratify tumor biospecimens of different grades.

**Results:**

We have developed a data-driven methodology for tumor classification using the profiles of CNAs reported by low-coverage sequencing. The proposed method called Bag-of-Segments is used to summarize fixed-length CNA features predictive of tumor grades. These features are further processed by machine learning techniques to obtain classification models.

High accuracy is obtained for classifying ovarian serous carcinoma into high and low grades based on leave-one-out cross-validation experiments. The models that are weakly influenced by the sequence coverage and the purity of the sample can also be built, which would be of higher relevance for clinical applications. The patterns captured by Bag-of-Segments features correlate with current clinical knowledge: low grade ovarian tumors being related to aneuploidy events associated to mitotic errors while high grade ovarian tumors are induced by DNA repair gene malfunction.

**Conclusions:**

The proposed data-driven method obtains high accuracy with various parametrizations for the ovarian serous carcinoma study, indicating that it has good generalization potential towards other CNA classification problems. This method could be applied to the more difficult task of classifying ovarian serous carcinomas with ambiguous histology or in those with low grade tumor co-existing with high grade tumor. The closer genomic relationship of these tumor samples to low or high grade may provide important clinical value.

## Background

Defined as somatic gain or loss of DNA regions, Copy Number Alterations (CNAs) are reflective of genomic instability, frequently affecting functionally important genes, such as tumor suppressors and oncogenes. CNAs are also associated with the early onset of tumor. They include both deletions and amplifications of large or small genomic regions. Large scale CNA events involving whole chromosome or chromosome arms alterations are also referred as aneuploidy. Small deletion events may target local regions of the genome harboring tumor suppressor genes locations, while amplifications preferentially target oncogenes locations [1]. As the consequence of tumor progression and evolutions, CNAs are not randomly distributed across the genome. The profiles of CNAs may provide a fingerprint specific to a tumor type or tumor class [2]. The recurrent CNAs across tumor types have been studied in an attempt to gain a deeper understanding of the pan-cancer mechanisms driving tumorigenesis.

Ovarian serous carcinomas, previously felt to be a disease continuum with a spectrum of differentiation from well to poorly differentiated, are now classified into two distinct categories, low grade and high grade serous carcinomas, based on their histologic features. These two groups are thought to be largely mutually exclusive based on their molecular characteristics. The majority (96%) of high grade serous carcinomas have TP53 mutations and show high levels of chromosomal copy number changes through the entire genome, whereas low grade serous carcinomas do not have TP53 mutations, show KRAS and BRAF mutations and in most cases are near diploid [3]. Additionally, low grade serous carcinomas have a more indolent prognosis and respond less well to standard platinum-based chemotherapy than high grade serous carcinomas [4].

The availability of Next Generation Sequencing (NGS) technology platforms has enabled the study of CNAs at a genome wide scale and at an unprecedented level of resolution. Not only the precision of the CNAs detection is enhanced but also the number of copy changes can be more accurately defined. Numerous methods are available to report CNAs from high-coverage whole genome sequencing and for low-coverage sequencing (LC-WGS). LC-WGS has recently gained interest since successfully translated into clinical applications. The Non-Invasive Prenatal Test (NIPT) is one example where cell free DNA of pregnant woman is sequenced at low coverage (<1×) to report the presence of fetal DNA aneuploidy. The expertise acquired in our group in the processing of LC-WGS has led us to explore how CNAs reporting from LC-WGS combined with machine learning techniques may be used to stratify tumor biospecimens of different grades.

Although for most cases the techniques such as evaluating the H&E and IHC for P53 expression status is helpful in determining the grade of serous carcinomas, pathologists found that these methods failed to provide conclusive results in cases with ambiguous morphology [5]. That can lead to ambiguity and difficulty in patient management. Here, we have introduced a data science methodology for tumor classification. The method can be used to assist the grade classification for the ovarian serous carcinomas, especially for the cases with ambiguous morphology.

The developed method called Bag-of-Segments (referring to CNA segments) is derived from the Bag-of-Features method. Bag-of-Features has been extensively used in the classification of image objects [6] and time series data [7]. Although currently surpassed by other methods such as deep learning, Bag-of-Features remains an ideal approach when dealing with small sample sizes like in the case for our study.

The Bag-of-Segments was used to obtain a fixed-length data representation of CNA segments that vary in numbers between samples. This fixed representation is needed for further processing of these features with machine learning techniques. The Bag-of-Segment approach was used to generate features needed for the development of a classification model for grading of ovarian serous carcinoma and was trained on CNA segments of 14 high-grade and 20 low-grade carcinoma samples. The CNA segments were derived from the LC-WGS data of these samples. The analysis of the Bag-of-Segment features contributes to the differentiation highlighted in two different underlying biological processes, one that involves large scale deletions or amplifications suggesting abnormal mitotic events, while the other involves local amplification and deletions commonly associated to DNA repair malfunctions.

## Method

The methodological approach includes several steps: 1) the processing of the low coverage sequencing data and reporting CNAs using an in-house developed tool, 2) the application of the Bag-of-Segment method to extract predictive CNA patterns and 3) the training of a classification model to predict the histologic type (low or high grade) of a sample.

### Patient samples

**Fig. 1:**
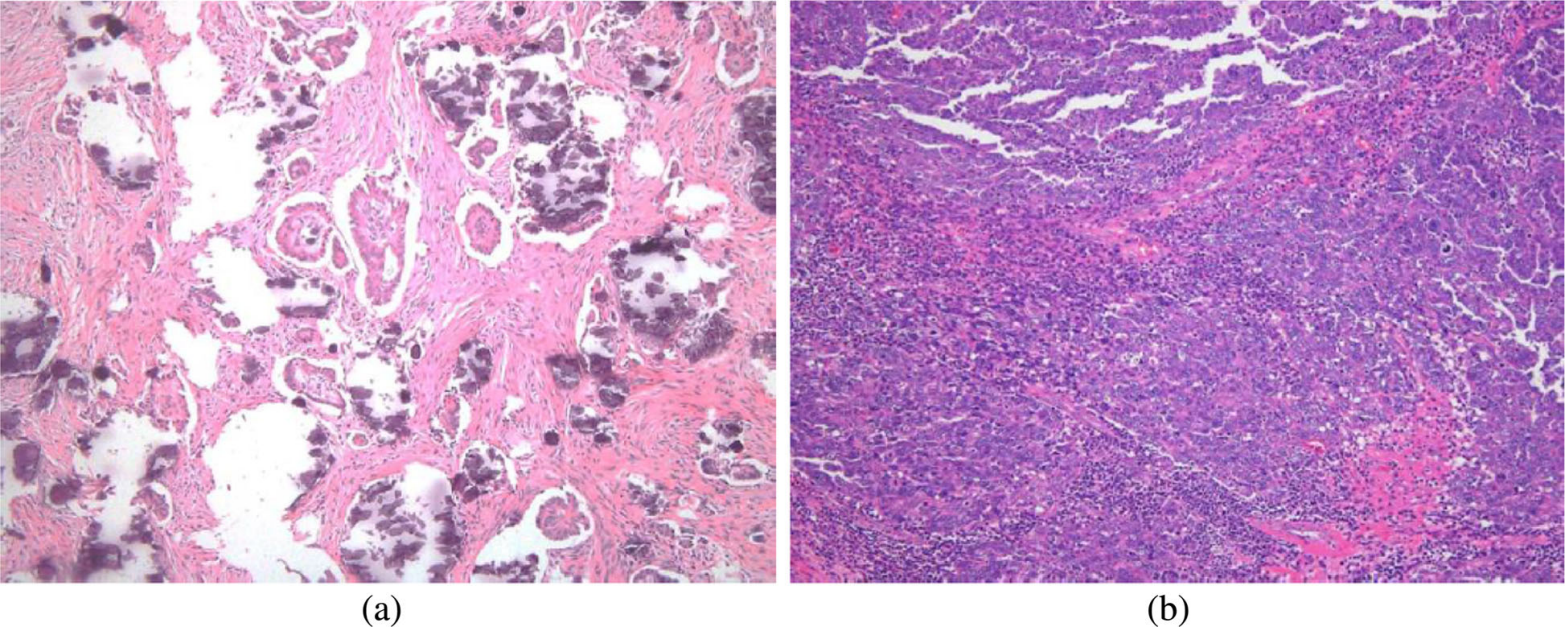
Photomicrograph of low grade and high grade serous carcinoma cases. **a** Low grade serous carcinoma, 20X. **b** High grade serous carcinoma, 20X

In this study, we collected and processed 34 sequencing coverage profiles from patients with ovarian serous carcinoma. Among these patients, 14 cases were diagnosed with high-grade and the remaining 20 with low-grade ovarian serous carcinoma based on the histologic review of the surgical material from tumor debulking surgery. The photomicrograph of low grade and high grade serous carcinoma examples is shown in Fig. 1. All cases were reviewed by a gynecologic pathologist. The MD Anderson two-tier classification system was used to classify ovarian serous tumors into low grade and high grade groups. We also used P53 immunohistochemistry in all cases for diagnosis confirmation. In each case, area of tumor was macrodissected from the Formalin-Fixed-Paraffin-Embedded (FFPE) tissue blocks with a minimum of 20% tumor cellularity, and DNA was extracted using Qiagen extraction kit. Sequencing reads were produced by Hiseq 4000, with multiplexing 8 samples per lane. The per-sample base-pair coverages range from ∼1× to ∼3×.

### LC-WGS preprocessing and CNA reporting

Samples were pre-processed with an in-house developed tool called Wandy [8]. Wandy accumulates the sparse sequence reads into 10,000 base long bins and performs several noise reduction procedures to more accurately characterize changes in coverage characteristic of Copy Number Variations (CNVs). As a result, each point in the input sequencing data is the coverage of WGS in 10kb genomic window. Wandy uses a top-down regression tree (CART algorithm [9]) to segment and identify the step-wise changes in the sequencing data. More specifically, the 1D regression tree model is fitted to the sequence coverage of each chromosome. To obtain a step-wise signal of a proper level of complexity, the CART algorithm is tuned by modifying the cost-complexity parameter (*Cp*). This parameter is control by the user and can be adjusted as a function of the need of the project. The selection of the optimal *Cp* value is described in a following section.

### Bag-of-Segments

**Fig. 2:**
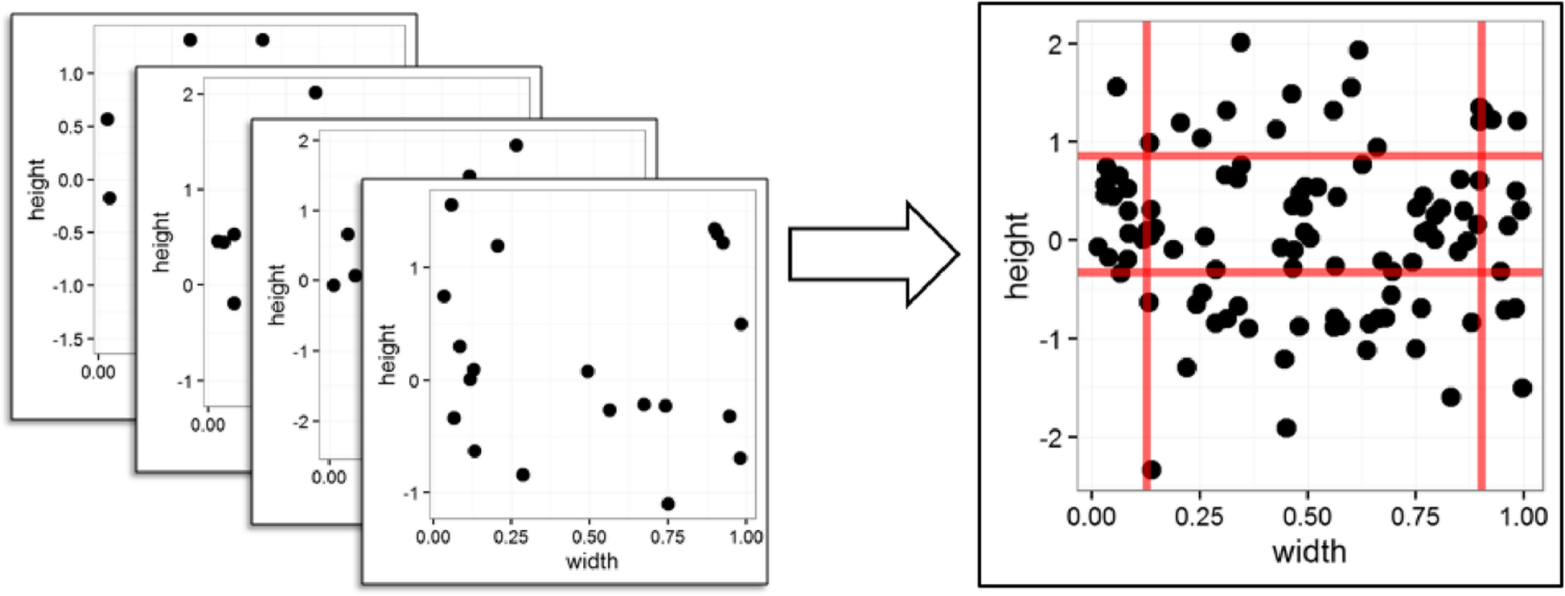
Bag-of-Segments representation workflow

The Bag-of-Segments approach used for this project is derived from the bag-of-features methodology [6, 7] and implemented as follow: first, each CNA segment is described by its height and width. The height is measured as the log2 ratio to the median coverage of the sample, and the width is measured in the proportion of the chromosome length. In our preliminary study, we observed that both the proportion of chromosome length and the actual length resulted in good classification accuracy. The normalization by the proportion of chromosome length is used to make CNV events more comparable across chromosomes. Then, CNA segments from all the samples are aggregated to produce a single 2D distribution of segment height and segment width, as illustrated in Fig. 2. The Bag-of-Segments is used to summarize the CNA profile of a sample in a limited set of features comparable across samples.

Let *h*_*α*_ and *h*_1−*α*_ denote the *α* and 1 - *α* percentiles of the segment heights, and let *w*_*α*_ and *w*_1−*α*_ denote the *α* and 1 - *α* percentiles of the segment widths. The red lines in Fig. 2 indicate these quantiles. Using the quantiles of both the width and the height, 9 CNA segment classes were defined as shown in Table 1. For each individual sample, the empirical frequency distribution of its segments over these 9 classes generates the Bag-of-Segments representation.

**Table 1:** Categorization of CNA segment using the adjusted quantiles of segment width and segment height

	*w*<*w*_*α*_	*w*_*α*_≤*w*<*w*_1−*α*_	*w*≥*w*_1−*α*_
*h*≥*h*_1−*α*_	Narrow Amplified (NA)	Medium Amplified (MA)	Wide Amplified (WA)
*h*_*α*_≤*h*<*h*_1−*α*_	Narrow Normal (NN)	Medium Normal (MN)	Narrow Normal (NN)
*h*<*h*_*α*_	Narrow Deleted (ND)	Medium Deleted (MD)	Narrow Deleted (ND)

### Parameter adjustments

The *Cp* and *α* are two parameters that respectively control the complexity of the CNA segment landscape and CNA segment classes defined by the bag-of-segment approach. These two parameters are set by the user and are adjustable as function of the sequencing coverage, complexity of the genomics alterations, quality of the sequencing results that is largely depending on the quality of the starting material (sample degradation, contamination and DNA amount). For instance, if *Cp* is set to a too small value, the segments could be adjusted to fit the noise in the sequencing results. On the opposite, a too large of *Cp* may not properly capture the biological signal. A proper choice of *Cp* is a trade-off between capturing the real signal and avoiding noise overfitting.

For this project and due to the small sample size, we used leave-one-out cross-validation (LOOCV) approach to adjust these 2 parameters. Sensitivity analysis was performed with different *Cp* values set to 0.001, 0.005, 0.01, 0.05, 0.1 and 0.2, and different *α* values set to 0.1, 0.15, 0.2, 0.25, 0.3, 0.35, 0.4 and 0.45. For each combination, the average accuracy of the Random Forest (RF) model (discussed in the next section) was obtained by repeated 10 times of the LOOCV.

### Classification model

A model was developed to classify samples into low-grade and high-grade serous carcinomas. We use a Random Forest (RF) [10], an ensemble model trained on the 9-feature Bag-of-Segments representation. Ensemble methods combine the predictions from many weak learners to present a stronger model. The condition for an ensemble model to outperform their individual members is the individual members are accurate (better than random guessing) and diverse (less correlated). RF is an ensemble of decision tree models each trained on a bootstrap sample of the original training data. A random subset of features is considered at each node split of each decision tree to make the weak learners even more diverse. As a result, RF usually presents a strong classification performance with less overfitting.

In addition to the strong performance, RF provides two benefits: first, it provides a continuous probability score for each sample indicating how likely the sample is high-grade by counting the vote proportion from the tree models. Second, the Gini importance score typically used in RF model enables the identification of the important features. More specifically, at each split of a node in fitting each tree model, the Gini impurity [11] is calculated from the two child nodes should be smaller than that of the parent node. For each variable, an importance score can be calculated by adding up the Gini deceases when it is selected over all the trees [10].

For the implementation, we used the R package “RandomForest” [12] for training the RF model with the default setting. By default, a RF model consists of 500 unpruned decision trees, and 3 features (square root of the total number of features) are randomly selected for the evaluation at each node split.

## Results

### Sample processing

**Fig. 3:**
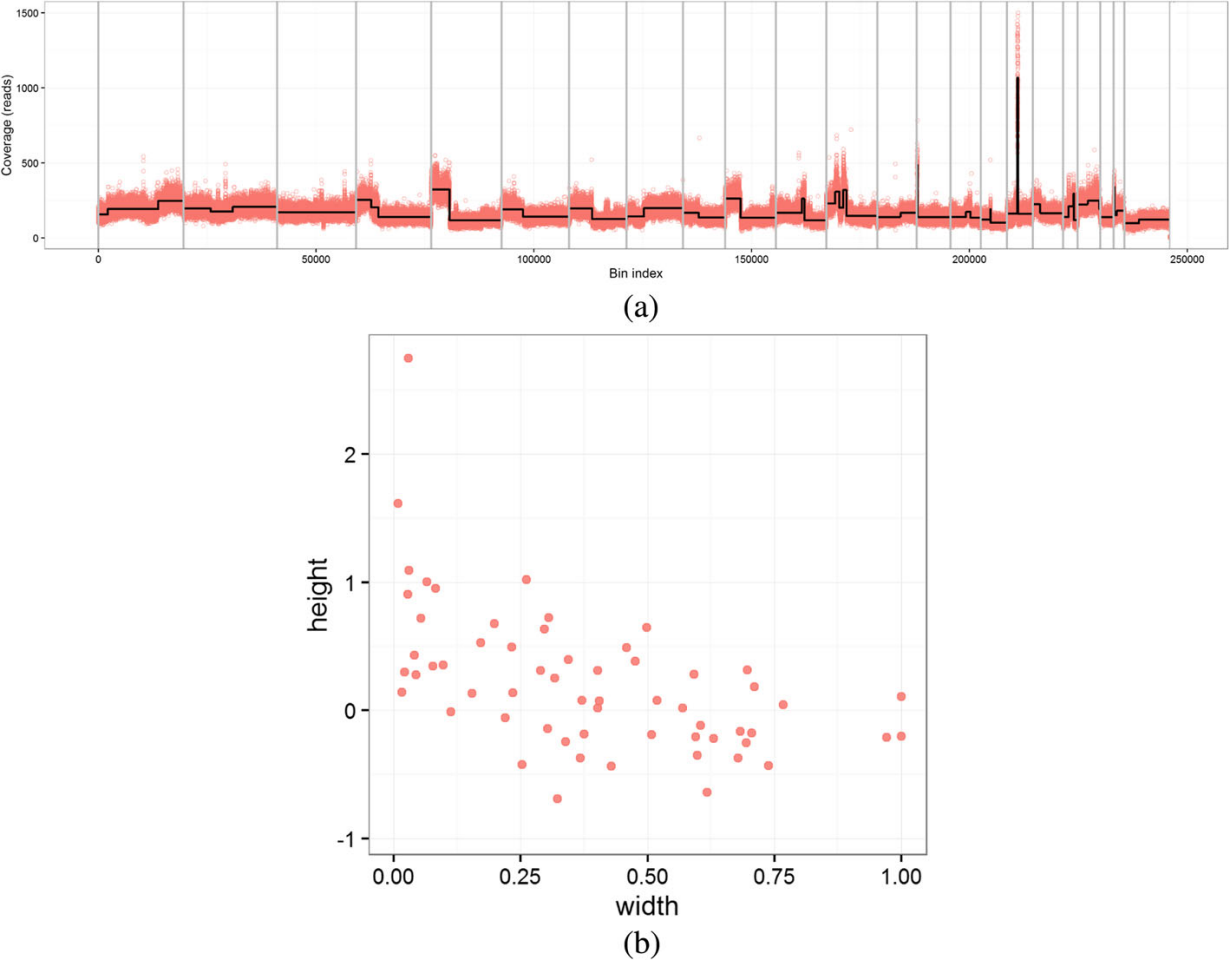
**a** Segmentation example for a CNA profile sample (23 chromosomes). **b** 2D distribution of the segment width and height for the segmentation in **a**

*Cp* = 0.05 and *α* = 0.25 were identified as one of the good performing settings and were selected to generate the data used by the classification model. For example, Fig. 3 gives the segmentation results from one sample with *Cp*=0.05. The corresponding 2D distribution of the segment width and height is shown in Fig. 3.

### Bag-of-Segments

**Fig. 4:**
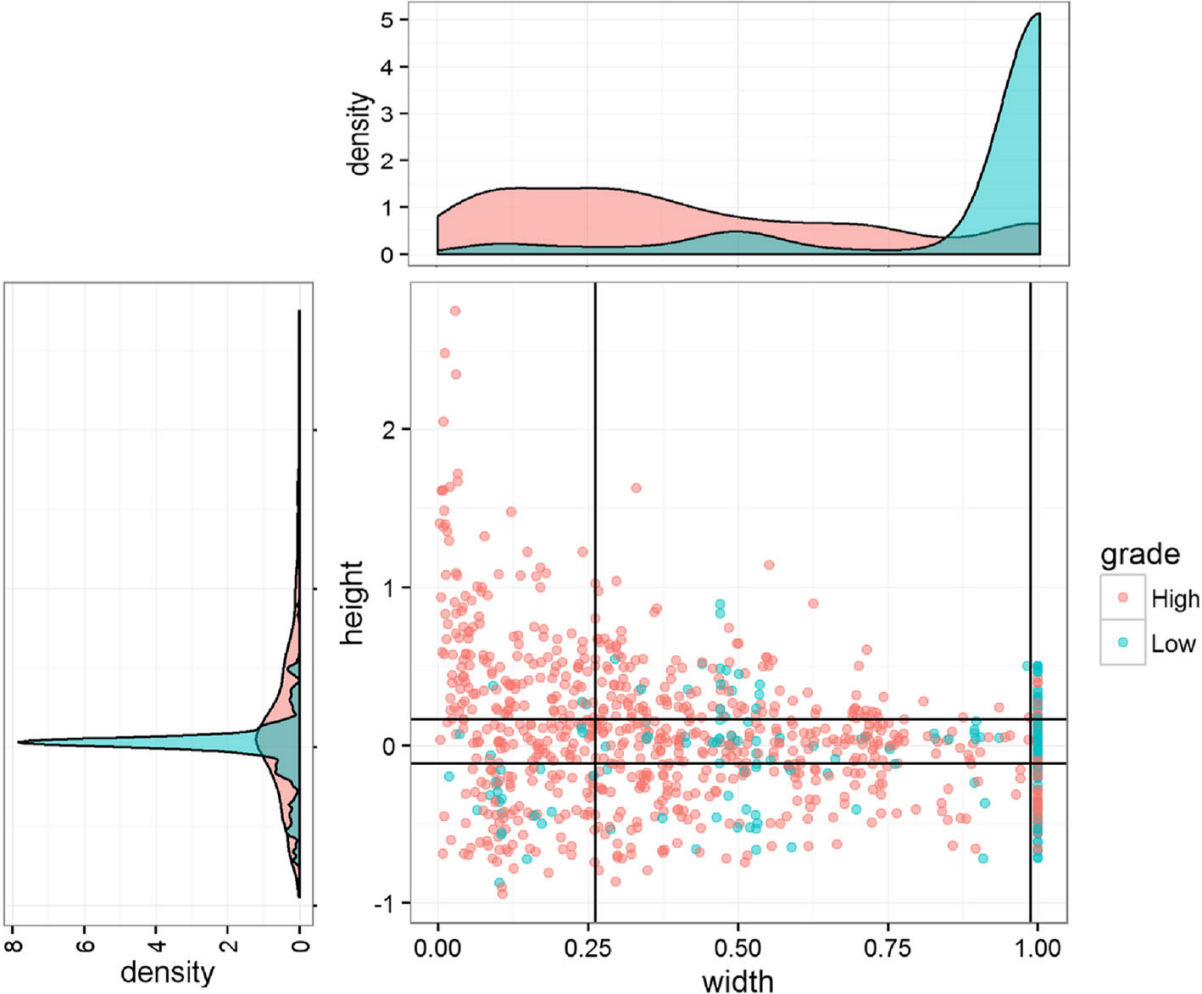
Aggregated joint distribution and marginal distributions of segment widths and heights

As shown in Fig. 4, the 2D joint distribution of the segment height and width as well as their marginal distributions are obtained after the aggregation over the 34 samples. The segments from the high- and low- grade samples are colored in red and blue respectively. From Fig. 4, we can easily observe that segments from the low grade- and high grade- samples follow different joint and marginal distributions in terms of their height and width. The results of the two-sample Kolmogorov-Smirnov tests that are provided in Table 2 further confirm our observation. The width distribution is more different than the height distribution based on the magnitude of the p-values. This observation lays the foundation of our bag-of-segment representation. We use the quantiles to discretize the continuous height-width space, so that we can use a fixed number of features to describe the joint distribution which is discriminative for the classification.

**Table 2:** Test results of the two-sample Kolmogorov-Smirnov tests for the segment width and segment height

Variable	D statistics	*P* value
Segment height	0.154	7.75×10^−7^
Segment width	0.702	<2.2×10^−16^

In Fig. 4 the quantiles (*α* = 0.25) of the segment height and the segment width are indicated by the black horizontal and vertical lines, accounting 9 CNA segment classes. The Bag-of-Segments representation is obtained based on the frequency distribution over the segment classes as shown in Table 3. This representation is used as the input of the RF model.

**Table 3:** Bag-of-Segments representation based on the distribution over the CNA segment classes

	MA	MD	MN	NA	ND	NN	WA	WD	WN	Grade
1	0.18	0.32	0.18	0.18	0.02	0.04	0.04	0.02	0.02	High
2	0.04	0.04	0.07	0.04	0.07	0.00	0.04	0.04	0.67	Low
3	0.00	0.14	0.07	0.00	0.07	0.04	0.14	0.11	0.43	Low
4	0.00	0.00	0.04	0.00	0.04	0.00	0.13	0.00	0.79	Low
...	...	...	...	...	...	...	...	...	...	...

### Parameter selection and sensitivity analysis

**Fig. 5:**
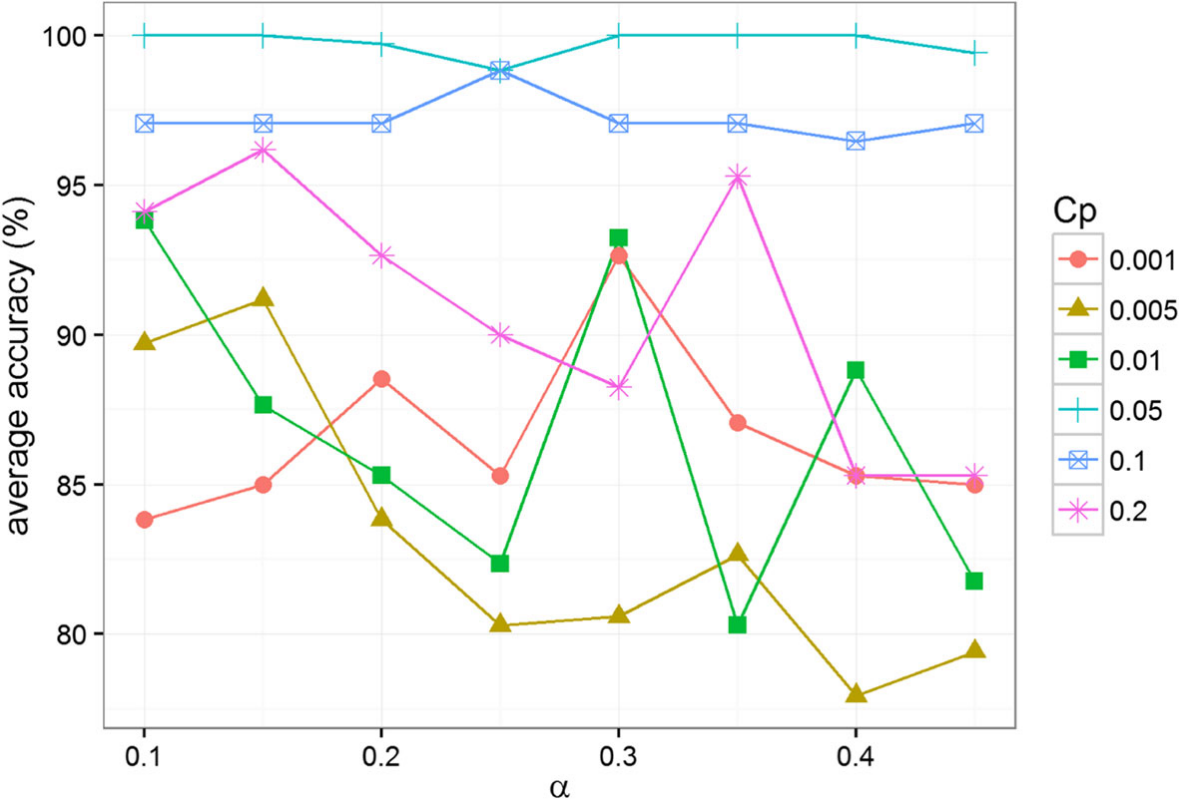
Sensitivity analysis with various *α* and *Cp* values

The *α* and *Cp* values were adjusted using LOOCV. Figure 5 gives the average LOOCV accuracies over 10 replicates of experiments for different selections of *α* and *Cp* values. Most parameter combinations achieve over 80% LOOCV accuracy, showing that the overall performance of our method is not very sensitive to the parameter settings. Moreover, over 98% LOOCV accuracy was obtained in various parameter combinations. It is shown that our method performs the best when *Cp* = 0.05. It achieved 100% accuracy with multiple different *α* values. When *Cp* is properly selected (= 0.1 or 0.05), our method works well with a wide range of *α* values.

## Discussion

### Model accuracy

Our method shows high accuracy in the ovarian serous carcinoma classification study. Based on the average LOOCV accuracy over 10 replicates, the accuracy of our method is close to 100% (99%) when setting *Cp* = 0.01 and *α* = 0.25 (the quarter quantiles are used for defining CNA segment classes for the simplicity). Although of high accuracy, we do recognize that the limited number of samples does not allow for an intensive testing of performance of the model. However, as discussed in the next section we believe that it should perform well on new samples.

**Fig. 6:**
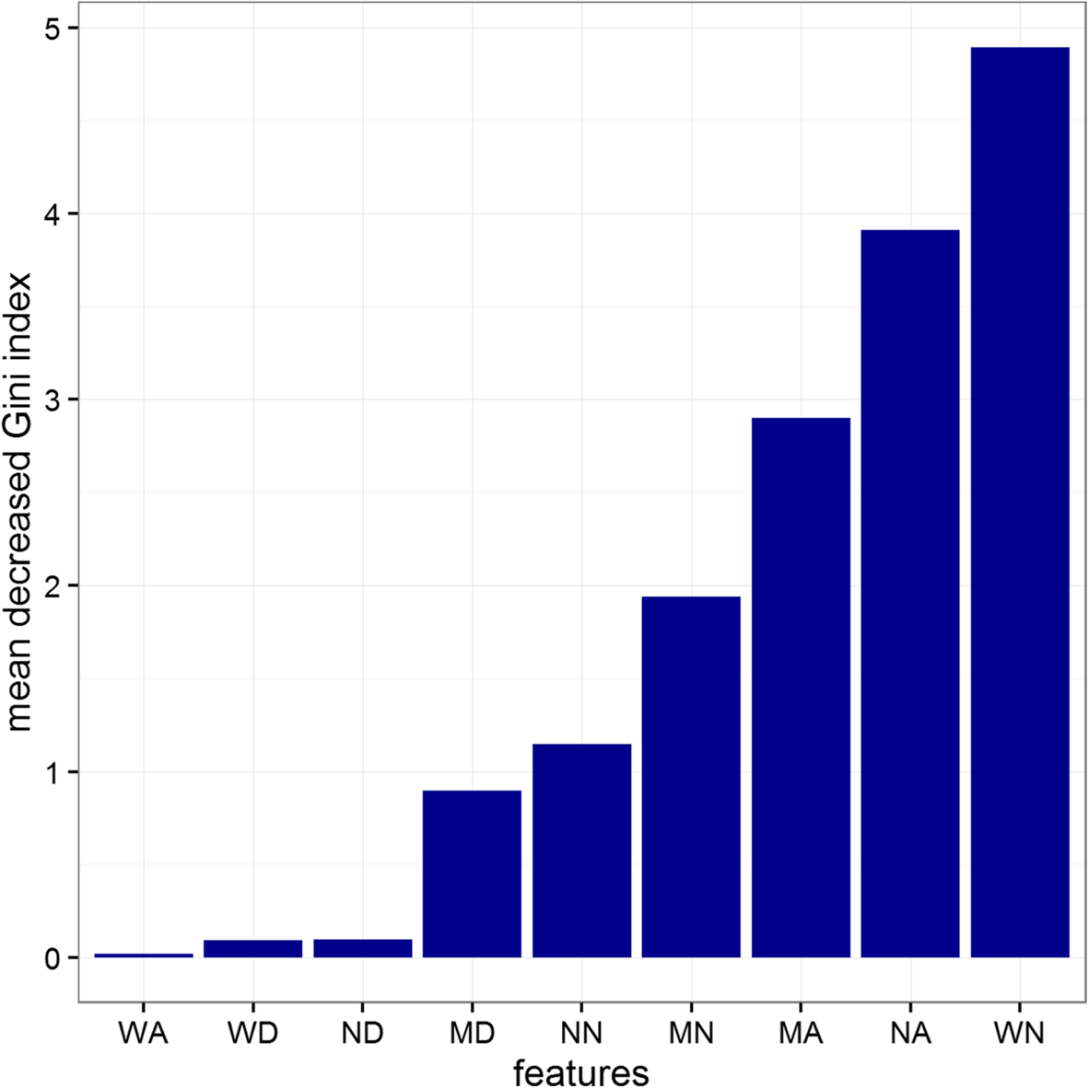
RF importance score for Bag-of-Segments features

**Fig. 7:**
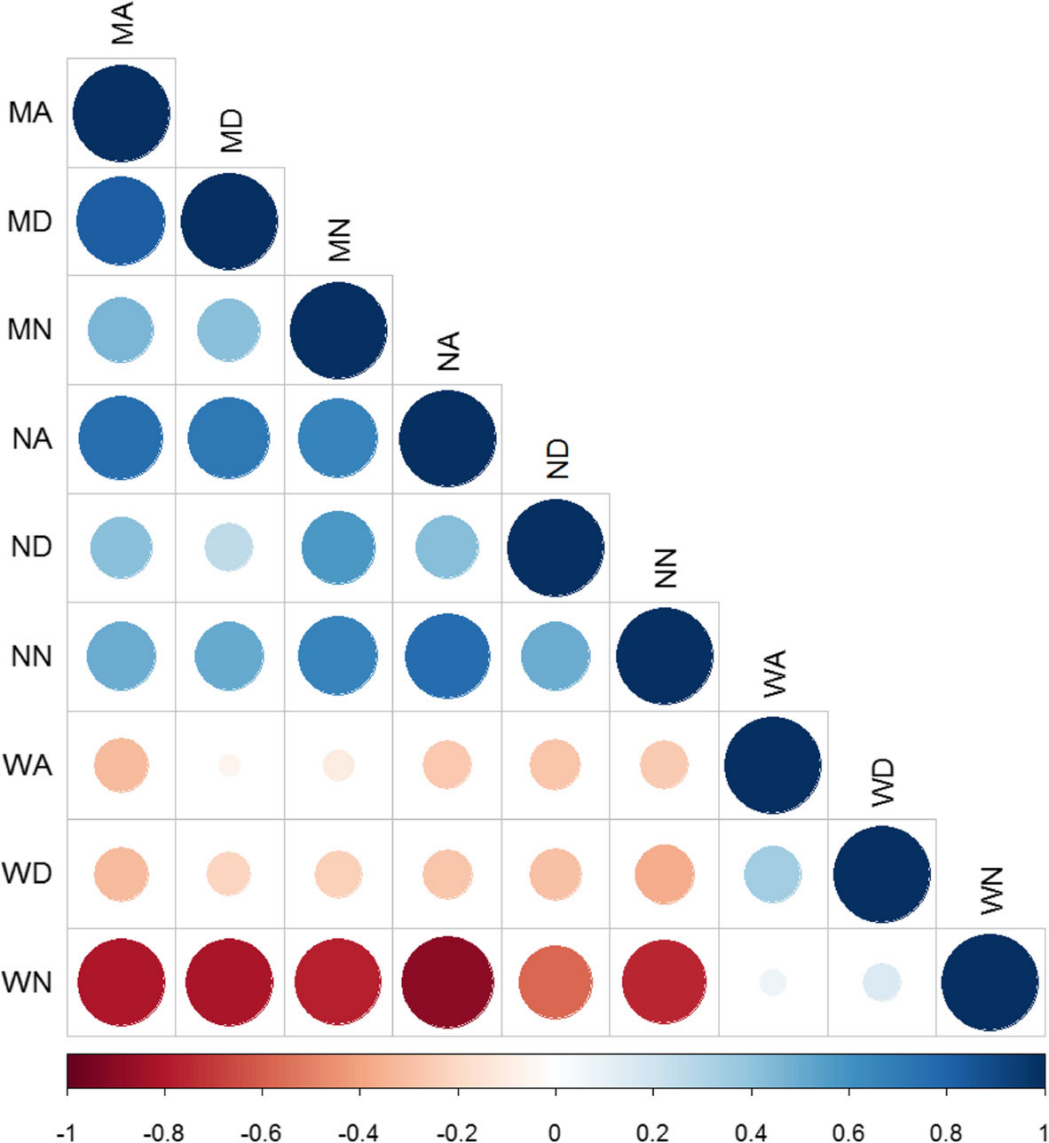
Correlation plot for Bag-of-Segments features

The two most important CNA segment classes identified by the Gini importance score are the Narrow Amplified (NA) and Wide Normal (WN) as shown in Fig. 6, although as displayed in Fig. 7, a high correlation exists between the 9 features used to build the model. This suggests that models with even lower dimensionality could be built. We investigated the independent contribution of the 2 dominant classes (NA, WN) by performing another set of 10 fold LOOCV using the same RF model. The model displays an average accuracy near to the previous one (99%) highlighting that these two features are sufficient for capturing the determinative information from the data.

### Generalization of the model

We expected the risk to overfit the model to be low since the Bag-of-Segments representation includes only 9 features, which is a very small compared to the dimensionality of the original data (over 25 thousand). The RF modeling, as an ensemble approach, also helps reduce the risk of overfitting as it takes the average of multiple models. Finally, the good performance of the model can be obtained by multiple parameter settings and is weakly affected by the small parameterization changes, also an indicator of low risk of overfitting and good generalization.

### Model Interpretation and clinical relevance of the most significant classes

**Fig. 8:**
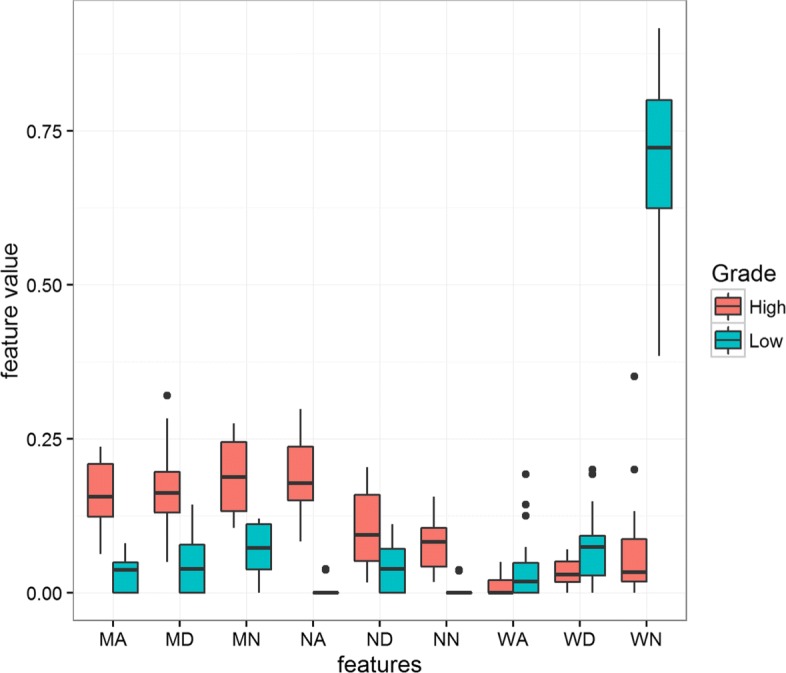
Box plots for the values of Bag-of-Segments features

The two most significant classes identified by the Gini importance score actually represent two groups of CNA classes. By reviewing the correlation plot in Fig. 6 we observe that the NA class is positively correlated with CNA segment classes ND, NN, MA, MD and MN. These classes represent narrow- or median- length CNA segments (group 1). The second class, WN, is positively correlated with WA and WD, a group of CNA classes associated to wide-length CNA segments (group 2). From the boxplot in Fig. 8, we can observe that CNA segments classes in group 1 are more frequent in the high-grade samples, than the low-grade samples which are more dominated by segment classes in group 2. Interestingly, these two groups of CNA classes represent two underlying biological mechanism of genomic instability. The first group is related to local amplification and deletion events often associated to DNA repair malfunctions. The second group represents large scale deletion or amplifications, such as the chromosome or arm deletion that are associated to errors taking place during the cellular division.

### Clinical applicability

The model in its current state has potential clinical value since it is highly specific and sensitive. However, the significant contribution of the height related group of CNA segments to the model could be of concern since the height related features are more affected by the sequencing coverage. Although the coverage was normalized across the genome, the height of amplified regions is dependent on the ratio of normal/tumor cells in a biological sample. This ratio may vary significantly as a function of the type of tissue biopsied, possibly affecting the performance of the model. In a attempt to reduce the influence of sequence coverage on the mode, we reduced the segment classes from 9 to 3, by only considering width-dependent features: Wide (W), Median (M) and Narrow (N) that are defined only by the quantiles of segment widths *w*_*α*_ and *w*_1−*α*_. This representation may also be considered as a linear combination of the original Bag-of-Segments representation, for example feature *W* = WA + WN + WD, and similar relationship is applied for feature M and feature N. We performed a 10 fold LOOCV using these 3 classes with *α* set to 0.25 and *Cp* set to 0.05. The average accuracy of the mode was 100%, suggesting that width-based features only can be used to accurately classify our ovarian dataset and therefore could be used to support clinical decisions. However, more samples are needed to validate this conclusion.

### Future work

This work proposes a data-driven alternative method for extracting patterns and classifying ovarian serous carcinomas. The features we extracted may be complementary to some known biological features such as Loss of Heterozygosity (LOH). However, calling LOH from LC-WGS is difficult since 1) the number of SNP positions that are sequenced is low and 2) most frequently only one SNP allele will be called from the sequencing data. Note that we are pursuing investigations in this space, exploring the use of imputation [13, 14] to help calling LOH.

Although our method has shown high accuracy on the 34 samples, we are actively working on collecting more samples, especially the ones with ambiguous morphology. In the future research, the analysis on more samples will help provide better understanding of the utility of this method in classification of ovarian serous carcinoma, especially those with challenging morphology and immune-profile.

## Conclusions

In this manuscript, we describe a new data-driven approach to classify ovarian serous carcinoma into high grade and low grade types with high accuracy. The proposed Bag-of-Segments method is used to summarize the CNA features from sequencing coverage data. The Bag-of-Segments was used to derive 9 features predictive of tumor type. The model obtained high accuracy with various parametrizations, indicating that it has good generalization potential towards other CNA classification problems. We recognize that more tumor samples are needed to fully investigate the predictive power of this model.

Due to the high correlation between several of 9 features, models of lower dimensionality could be built. We demonstrated that Narrow Amplified (NA) and Wide Normal (WN) CNA features were sufficient to discriminate low grade from high grade ovarian tumor samples. NA and WN features represents two groups of CNA classes respectively. The patterns captured by these two groups correlate with current clinical knowledge: low grade ovarian tumors being related to aneuploidy events associated to mitotic errors while high grade ovarian tumors are induced by DNA repair gene malfunction.

We also have shown that models independent from high coverage could also be successfully built. Beyond methodological interest, this result indicates that models that are weakly influenced by the sequence coverage and the purity of the sample defined by the ratio tumor/normal cell in a sample can be built. These models would be of higher relevance for clinical applications.

Finally, we believe that this new method could be applied to the more challenging task of classifying ovarian serous carcinomas with ambiguous histology or in those with low grade tumor co-existing with high grade tumor. We are collecting these morphologically challenging ovarian serous carcinoma cases, and by modeling the low grade and high grade serous carcinoma, we hope to be able to characterize their molecular nature and have a better understanding of their pathogenesis. Classification of ovarian serous carcinomas to low or high grade based on their genomics may provide valuable clue on how to best manage these patients in clinic.

## Abbreviations

CNA: Copy number alternation
CNV: Copy number variation
CART: Classification and regression tree
Cp: Cost-complexity parameter
FFPE: Formalin-fixed-paraffin-embedded
LC-WGS: Low-coverage whole genome sequencing
LOOCV: Leave-one-out cross-validation
NGS: Next generation sequencing
NIPT: Non-invasive prenatal test
RF: Random forest
